# Advanced Cardiac Imaging for Risk Prediction of Pacing-Induced Cardiomyopathy: A Narrative Literature Review

**DOI:** 10.3390/jcm15041358

**Published:** 2026-02-09

**Authors:** Karla Asturias, Sarah Li, Shivani Reddy, Peter M. Jessel, North J. Noelck, Bhaskar Arora, Mahi Lakshmi Ashwath, D. Elizabeth Le

**Affiliations:** 1 Knight Cardiovascular Institute, Oregon Health & Science University, Portland, OR 97239, USA; asturiak@ohsu.edu (K.A.);; 2 Department of Internal Medicine, Baylor University Medical Center, Dallas, TX 75246, USA; shivani.reddy@bswhealth.org; 3 Division of Cardiology, Veterans Administration Portland Health Care System, Portland, OR 97239, USA; 4 UHS/UT Heart and Vascular Institute, San Antonio, TX 78240, USA

**Keywords:** right ventricular pacing, pacing-induced cardiomyopathy, echocardiography, cardiac CT, cardiac MRI

## Abstract

**Background/Objective**: Pacing-induced cardiomyopathy (PICM) is a common complication of right ventricular (RV) pacing, affecting 6–25% of patients with frequent RV pacing, due to electrical and mechanical dyssynchrony. Certain clinical and electrocardiographic risk factors have been identified, including high RV-pacing burden and longer paced QRS, but their ability to predict the development PICM remains limited. Additionally, other forms of PICM have been described, including heart failure with preserved ejection fraction and RV failure. The goal of this narrative review is to summarize the current evidence utilizing noninvasive imaging to identify patients predisposed to a high risk of PICM. **Methods**: Using a literature search in the PubMed, Scopus, Google Scholar, and the Cochrane databases from 2000 to 2025, which included but was not limited to the keywords right ventricular pacing, pacemaker-related cardiomyopathy, pacemaker-induced cardiomyopathy, biventricular pacing, conduction system pacing, His bundle pacing, left bundle pacing, echocardiography, computed tomography imaging, and cardiac magnetic resonance imaging, we reviewed randomized control trials, observational retrospective and prospective cohort studies, societal guidelines, and systematic review articles. **Conclusions**: Essential in the diagnosis of PICM, cardiac imaging can identify patients at risk, even before left ventricular (LV) dysfunction or symptoms develop. Pre- and early post-implantation 2- and 3-dimensional echocardiography with global longitudinal strain provides sensitive parameters for the potential development of PICM. Relative indices of contractile asymmetry have been described. Cardiac magnetic resonance imaging offers an accurate assessment of cardiac volumes and LV synchrony and can also quantify myocardial fibrosis, a significant predictor of PICM. Performing pre-device implantation imaging may help predict subsequent heart failure development and potentially can guide pacing modality selection that can mitigate this risk. Thus, an imaging-guided framework will advance the management of PICM.

## 1. Introduction

Pacing-induced cardiomyopathy (PICM) is a common clinical entity resulting from electrical and mechanical dyssynchrony due to frequent right ventricular (RV) pacing. Despite affecting up to 6–25% of patients with RV pacing, there is no consensus on its definition, and the most optimal and cost-effective management strategy remains undefined [1,2]. PICM treatment has focused on biventricular synchrony recovery with cardiac resynchronization therapy (CRT) or conduction-system pacing (CSP) [3], which includes left bundle area pacing (LBAP) and His bundle pacing (HBP). Reported risk factors for PICM include the longer paced QRS duration, lower baseline left ventricular ejection fraction (LVEF), baseline left ventricular (LV) end-systolic dimension, and high RV pacing burden [4,5,6]. However, risk prediction remains uncertain, and vulnerable patients have not been adequately identified. In an era with more widely available advanced imaging, there have been multiple efforts to identify patients who are most at risk with two-dimensional echocardiography (2DE), three-dimensional echocardiography (3DE), speckle-tracking echocardiography (STE), computed tomography (CT), and magnetic resonance imaging (MRI). In this narrative review, we will summarize the current evidence supporting utilization of advanced cardiac imaging to identify patients at a higher risk of PICM.

## 2. Methods

We performed a literature search in the PubMed, Cochrane, Google Scholar, and Scopus databases from 2000 to 2025 using the following but not limited to terminology, phrases, or combination of phrases: definition, epidemiology, incidence, risk factors, pathophysiology, medical therapy, pharmacotherapy, pacemaker, cardiac pacing, right ventricular pacing, permanent pacing, dual-chamber pacing, biventricular pacing, conduction system pacing, His bundle pacing, left bundle pacing, pacemaker-induced cardiomyopathy, pacing-induced cardiomyopathy, pacing cardiomyopathy, pacing-related cardiomyopathy, right ventricular pacing-induced dysfunction, dyssynchrony-induced cardiomyopathy, RV pacing-related cardiomyopathy, echocardiography, echocardiogram, strain imaging, speckle tracking, global longitudinal strain, three-dimensional echocardiography, cardiac magnetic resonance, late gadolinium enhancement, T1 mapping, native T1, T2 mapping, extracellular volume, fibrosis, myocardial scar, myocardial edema, myocardial inflammation, tissue characterization, cardiac CT, computed tomography, advanced cardiac imaging, and myocardial deformation. Inclusion criteria included but were not limited to clinical studies published in the English language, randomized controlled trials, observational retrospective and prospective cohort studies, research letters and correspondence, participants age ≥ 18 years recruited from any racial/ethnic population groups, the most recent societal guidelines, and systematic review articles. Initial search with the inclusion criteria produced 991 publications (580 PubMed, 20 Cochrane, 330 Google Scholar, and 61 Scopus). Review of the abstracts associated with these manuscripts yielded 186 relevant articles. The findings from these publications were summarized to provide a concise presentation of the pathophysiology of PICM, clinically available pacing modalities, therapies and interventions for PICM, and the role of multi-modality imaging in risk prediction and management of this category of cardiomyopathy. Since this work is not a systematic review, the limitations of this narrative review include selection bias, retrieval bias, language bias, and publication bias.

This study is a clinical narrative literature review and does not contain a collection of primary human data, and thus institutional review board approval, informed consent, and dataset availability or location are not applicable.

## 3. Background

PICM is defined as the reduction in LVEF in the setting of chronic and frequent RV pacing. The specific thresholds to diagnose PICM have varied significantly between studies [7], but the most common definition is a ≥10% decrease in LVEF, resulting in LVEF < 50% after pacemaker implantation [1]. Alternative definitions include LVEF < 40%, LVEF < 45%, decline in the LVEF ≥ 5%, or need for a CRT upgrade. Some studies consider the presence of ventricular dyssynchrony determined by an imaging modality, an important feature in the diagnosis of PICM [8]. The definition of high RV pacing is frequently not provided in studies, but, when available, an RV pacing burden of ≥20% is usually considered significant. However, other studies have designated thresholds of ≥40% [1].

Although PICM has been typically associated with LV dysfunction, pacemaker-induced RV cardiomyopathy (PI-RVCM) or biventricular phenotypes of cardiomyopathy have also been described. A case series of 127 patients reported an incidence of PI-RVCM of 11.0% at a median of 29 months following pacemaker implantation. Of those patients, 4% had biventricular dysfunction, and 7% had isolated RV dysfunction [9]. Pacing has also been associated with heart failure with preserved ejection fraction (HFpEF) phenotypes, isolated LV diastolic dysfunction, and mitral and tricuspid regurgitation due to mechanical dyssynchrony or direct interference of the pacemaker lead with the tricuspid valve [10].

In patients with normal LVEF and atrioventricular block (AVB) who are expected to have high RV pacing burden, the PICM incidence has been estimated to be 6–25%. This approximation, however, is highly dependent on the PICM definition, the specific population studied, and the lengths of follow-up [1,7,11]. The diagnosis of PICM has been reported between 1 month and 16.9 years after implant, with the majority being diagnosed 13 months to 5.2 years after pacemaker implantation [1]. With the increased utilization of conduction system pacing, the incidence of PICM appears to be decreasing in patients requiring frequent ventricular pacing, with reported incidences as low as 2.5–5.8% [12,13,14].

Even though a specific pathophysiology has not been fully elucidated, the primary mechanism of PICM development appears to be intimately related to interventricular dyssynchrony, a similar entity to left bundle branch block (LBBB) cardiomyopathy and premature ventricular contraction (PVC) cardiomyopathy [7,15]. With RV pacing, there is myocyte-to-myocyte propagation of the action potential; butfailure to activate the His–Purkinje system early can result in impaired mechanical contraction and delayed activation of the basal and lateral wall segments of the left ventricle [16]. There is also evidence that chronic and frequent RV pacing can be associated with a reduction in the expression of calcium channels in the myocyte, which can precede LV negative remodeling [17]. Additionally, alterations in myocardial perfusion, fatty acid metabolism, and changes in the autonomic nervous system have been described with RV pacing [18].

Multiple case series and cohort studies have consistently identified clinical and electrocardiographic risk factors for PICM that are summarized in Table 1. Pre-implant risk factors include older age [19], male gender [20], wider native QRS of >115 ms [3,11,21,22], lower pre-implant LVEF [7,19,23], and increased LV diameter [24]. After pacemaker implant, the RV pacing burden has been correlated with increased risk [8,23]. Additionally, the paced QRS duration of >150 ms, which is a surrogate for dyssynchrony, has also been found to be a reliable risk factor [4,8,9,20,22,24,25]. In a small prospective descriptive single center study of 27 patients who were developing PICM from RV pacing, ultra-high frequency electrocardiograms identified dyssynchrony and prompted upgrade from traditional to CSP. The average baseline LVEF improved from 34.5% (27–42%) to 47.6% (38.2–57%), *p* < 0.0001 in 17 patients who received HBP and in 10 patients who received LBAP [26]. Furthermore, in a meta-analysis of 3 studies with 28,525 patients, a history of myocardial infarction was a potential risk (odds ratio 1.81 [95% CI 1.54–2.12], *p* < 0.001). A meta-analysis of 2 studies with 28,322 patients demonstrated that chronic kidney disease was associated with PICM (odds ratio 1.66 [95% CI 1.32–2.01], *p* < 0.001), and a meta-analysis of 2 studies with 21,405 patients found that atrial fibrillation was also associated with PICM (odds ratio 1.32 [1.23–1.42], *p* < 0.001) [1].

**Figure 1:**
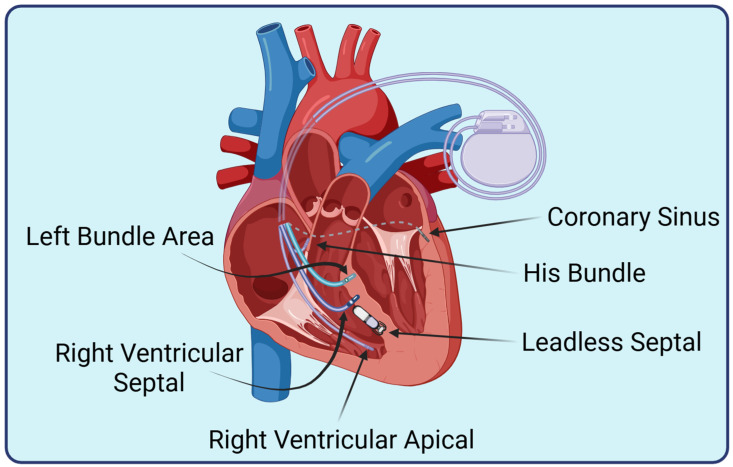
**Pacing modalities.** Depiction of lead implantation anatomic sites. Created in https://BioRender.com.

In addition to patient-specific factors, the risk for developing PICM is also influenced by the pacing modality itself. Traditional RV apical pacing (RVAP), RV septal pacing (RVSP), leadless pacing, CRT, and CSP produce differential effects on mechanical dyssynchrony and the development of cardiomyopathy (Figure 1).

## 4. Pacemaker Categories

### 4.1. Right Ventricular Pacing

Standard RV pacing may provide lifesaving therapy, but the non-physiologic activation pattern akin to a LBBB leads to a cascade of detrimental effects [1]. The RV apex was the favored site of endocardial pacing due to the trabeculae facilitating implantation of passive leads that were initially developed, with active fixation helix leads becoming available later [27].

RV septal pacing has been proposed to reduce the risk of PICM, although high quality data have been limited with several randomized controlled trials showing no difference in clinical events or LV function on echocardiogram [28,29]. The largest study, a propensity score matched cohort, similarly demonstrated no benefit in combined heart failure hospitalization and death [30]. It is worth noting that it may be difficult to achieve true RV septal lead position at the time of implant with standard stylets and usual fluoroscopic views alone [31]. A subsequent echocardiogram may reveal the lead tip is not in a septal position.

### 4.2. Biventricular Pacing

CRT was developed after the link between LBBB and LV dysfunction was more firmly established in the 1990s [32]. Unlike traditional forms of RV pacing, this therapy was shown to promote reverse remodeling [33]. To correct interventricular conduction delay, coronary sinus (CS) leads were developed to directly pace the epicardial surface of the LV. The first randomized clinical trial (RCT) completed in 2001, MUSTIC, showed a significant improvement in the 6 min walk test and quality of life, with subsequent trials demonstrating improvement in survival and reduction in heart failure hospitalization [34].

### 4.3. Leadless Pacing

The RV apex was initially the preferred site for leadless pacemakers; however, the Micra Investigational Device Exemption registry showed a perforation rate of 1.6% [35]. The subsequent post-approval registry transitioned the favored implant site to the RV septum, reducing the perforation rate by 4-fold to 0.4% [36]. Thus, leadless ventricular pacing has largely become a septal location while standard transvenous RV pacing remains apical. Several single center studies have shown conflicting results regarding the risk of PICM in leadless pacemakers, with the incidence ranging from 3% to 43% [37,38], which may be explained by variable definitions of PICM. A more recently published multicenter review showed no significant difference in PICM rate between groups, while QRS width was a predictor for PICM [39].

### 4.4. Conduction System Pacing

Physiologic pacing via the His bundle gained interest as an alternative to RV pacing and the less physiologic epicardial pacing via the CS, achieving a near normal ventricular synchrony [40]. However, elevated thresholds and the potential need for lead revision tempered its use, especially after demonstration of LBAP [41]. Deep penetration of the RV septum allows a right-sided lead to capture the left bundle with low thresholds, although confirming capture can be more challenging than the His bundle position [42]. This technique has demonstrated benefits in observational studies and small randomized control trials (RCTs) [43], but larger RCTs demonstrating the benefits in heart failure and potential superiority to CS pacing are pending [44].

## 5. Current Treatment for Pacing-Induced Cardiomyopathy

### 5.1. Medical Therapy

Pharmacotherapy plays a modest role in the prevention and treatment of PICM. Although device-based strategies remain the cornerstone of PICM management, emerging evidence suggests that guideline-directed medical therapy (GDMT) may reduce the risk of developing PICM in patients with a high RV pacing burden and may have a synergistic role with the device-based treatment of PICM, as summarized in Supplementary Table S1.

#### 5.1.1. Medical Therapy in the Prevention of Pacing-Induced Cardiomyopathy

A single center, retrospective study of 642 patients with normal pre-implant LVEF, who underwent pacemaker implantation for complete heart block and experienced substantial RV pacing burden (average of 80–86%), demonstrated a significant reduction in PICM incidence among patients receiving angiotensin-converting enzyme inhibitors (ACEIis) or angiotensin-receptor blockers (ARBs), β-blockers, or combination therapy. The incidence of PICM over 10 years was 4.7% in treated patients compared with 7.0% in untreated controls, corresponding to an adjusted hazard ratio (HR) of 0.59 (95% confidence interval (CI) 0.45–0.77). Combination therapy provided the greatest risk reduction (HR 0.46, 95% CI 0.31–0.69). The median time to PICM development was 4.7 years (interquartile range 3.2–7.1) [45]. These data support the use of ACEis, ARBs, and β-blockers as a preventive strategy in patients with anticipated high RV pacing burden.

#### 5.1.2. Medical Therapy in the Treatment of Established Pacing-Induced Cardiomyopathy

For patients with established PICM, pharmacotherapy with standard GDMT for heart failure is recommended [46,47]. However, no RCTs to date have evaluated medication-only therapy for PICM, and evidence indicates that GDMT alone is insufficient to reverse established PICM [3]. In a prospective cohort study of 615 patients with RV pacing ≥90% and PICM, optimal medical therapy alone did not lead to significant improvement in LVEF or New York Heart Association (NYHA) heart failure class at 1-year follow-up when compared with the group that received a CRT upgrade after at least 3 months of optimal medical therapy [48].

While pharmacotherapy is not recommended as a stand-alone treatment, the addition of GDMT to CRT is adjunctive and may be beneficial. A post hoc analysis of the prospective UPGRADE trial showed that the prior use of β-blockers, mineralocorticoid receptor antagonists (MRAs), ACEi, or ARB therapy did not reduce the therapeutic response to the CRT upgrade [49]. Another retrospective study of 43 patients with PICM undergoing a CRT upgrade found that ACEi and ARB therapy was associated with a trend of greater improvement in LVEF (+7.21%, *p* = 0.05), suggesting a possible synergistic remodeling effect through neurohormonal mechanisms even in the presence of pacing-induced mechanical dyssynchrony [50]. In addition to conventional GDMT, atorvastatin has been studied in patients with pacemaker implantation for high-grade AVB because there are data suggesting that pacing can induce lipotoxic cardiomyopathy. In a study of 1717 patients, statin treatment was associated with a significant lower risk of heart failure hospitalization, cardiovascular mortality, and all-cause mortality [51].

#### 5.1.3. Medical Therapy: Areas of Future Research

Further investigation is needed to determine whether contemporary agents such as angiotensin receptor–neprilysin inhibitors and sodium–glucose cotransporter-2 inhibitors can provide complementary benefits in the PICM population. These therapies may be particularly valuable for patients who are not candidates for a device upgrade or who exhibit incomplete reverse remodeling despite optimal pacing strategies.

### 5.2. Device Optimization

Programming a standard atrioventricular (AV) delay sufficiently long to allow for intrinsic conduction in patients without CRT is a basic tenet of device management. Following recognition of the deleterious effects of RV pacing, device manufacturers developed specific algorithms to reduce ventricular pacing percentage. The two main strategies include a progressive increase in AV delay (AV hysteresis) and promotion of atrial only pacing with mode switch algorithms (atrial pacing with intact conduction, back-up ventricular pacing with loss of AV conduction). Both are limited to patients with mostly intact AV conduction but may have prolonged PR intervals or brief periods of AV block that would otherwise result in RV pacing. Clinical guidelines continue to support minimizing unnecessary RV pacing [52], although studies evaluating the impact on actual clinical outcomes have been mixed. A recent systematic review and meta-analysis of pacing algorithms to minimize RV pacing in >7000 patients showed a 35% reduction in heart failure hospitalization and no increase in symptoms such as syncope [53].

### 5.3. Alternative Pacing Modalities

Although the upgrade of PICM patients to CRT has been well recognized to improve outcomes, the first RCT to prove this was the BUDAPEST-CRT Upgrade trial in 2023 [54]. Included patients had a pacemaker or implantable cardioverter defibrillator with RV pacing of ≥20% in the past 3 months and a paced QRS of ≥150 ms. The CRT upgrade group showed a reduction in composite of heart failure hospitalization, mortality, <15% reduction in LV end systolic volume from baseline, and, importantly, a similar benefit in patients with atrial fibrillation versus sinus rhythm. A large single center observational study showed similar improvement in heart failure outcomes and procedural complication rate in patients with de novo CRT compared to those with a later upgrade to CRT [55]. There was increased complexity in those with venous occlusive disease, requiring modification of the implant technique in 26% of these patients, but lead extraction for all upgrade procedures remained relatively low at 4.7%. Generally, the response rate for CRT in patients with a PICM diagnosis appears to be higher than in the general CRT population, 85–92% versus approximately 67%, respectively, as expected for a direct treatment of a heart failure etiology [50,56].

The first description of permanent HBP published in 2000 was performed in patients with atrial fibrillation and dilated cardiomyopathy, demonstrating this as a de novo therapy for heart failure [57]. LBAP pacing has mostly replaced HBP, although large trials are lacking to confirm the improvements in heart failure outcomes seen in observational studies, and data for PICM are even more limited. The largest study to date for LBAP pacing in PICM, with just 64 patients, showed a significant improvement in LVEF (+11 ± 17.7%, *p* < 0.001) and NYHA heart failure classification (2.72 ± 0.68 to 1.75 ± 0.6, *p* < 0.001). Even though complication rates were very low, approximately one-third of patients had a lead extraction at the time of upgrade [58]. It is reasonable to expect that LBAP will have high efficacy for PICM, which is similar to or better than CS lead-based CRT.

## 6. Noninvasive Imaging for Risk Prediction of PICM

### 6.1. 2D- and 3D-Echocardiography

2DE remains the imaging modality of choice to determine the risk factors of PICM [3]. It is essential in defining LV and RV anatomy and function, which can mitigate procedural challenges and complications. Due to its versatility, echocardiography has been utilized to guide lead placement in CSP, to customize specific anatomy and electromechanical indices, and to assess valvular dysfunction, which can contribute to PICM [59]. Identification of the prominent moderator band or septal trabeculations may alter pre-implantation planning as lead placement and implantation can be challenging.

Echocardiography-derived LV size and function are the fundamental metrics of PICM assessment. A baseline LV end-systolic diameter has been demonstrated to be an independent predictor of subsequent PICM and heart failure hospitalization (HR = 1.12, 95% CI 1.03 to 1.22, *p* = 0.008) in a study of 203 patients who were anticipated to have high RV-pacing burden at the time of pacemaker implantation [11]. In addition, severe LV dilation (LV end-diastolic diameter > 70 mm or LV end-systolic diameter > 60 mm) can result in inefficient or ineffective LBAP because the pacing stimulus may not reach the left bundle branch [60]. Pre-implantation LVEF [61] and LVEF < 50% as measured by 2DE [62] have been demonstrated to be independent predictors of developing PICM. Even with pre-implantation LVEF in the normal range, lower baseline LVEF also independently predicted PICM in a univariate analysis in a retrospective study of 277 patients [22].

In patients with conduction disease, the Simpson’s biplane method of calculating the LVEF is not as sensitive as tissue Doppler imaging to determine regional dyssynchrony. Even though physiologic pacing can reduce the risk of PICM, when it is suboptimal, cardiomyopathy can develop. In patients with CRT, echocardiography-derived LV volumes at end-diastole and end-systole and LVEF improvement at 12 months have predicted death and heart failure events. In the MADIT-CRT trial, every reduction of 10% in LV end-diastolic volume or a 5-point increase in LVEF was associated with a 40% reduction in death or heart failure hospitalization [63,64,65]. Similar outcomes in patients with LBAP and HBP have not been investigated, but in a prospective cohort of 151 patients treated with LBAP or CRT with median follow-up of 23 months (15.5–28 months), PICM did not occur in patients with preserved LVEF or with improved LV function in the context of reduced baseline LVEF [66].

3DE is superior to 2DE in the measurement of LV volume and can detect subtle alteration in left and right ventricular function and adverse ventricular remodeling [21,60]. Interestingly, in a small prospective cohort of 36 patients, 3DE-derived LVEF was associated with PICM both at 24 h and 6 months after pacemaker implantation [67]. Thus, when available, volumetric function assessment by 3DE is preferred.

RV function can also play an important role in the development of PICM. In a retrospective study of 127 patients, those who had lower RV tissue Doppler developed PI-RVCM compared with those who did not develop cardiomyopathy (11.8 cm/s versus 13.8 cm/s, *p* = 0.02). An incremental increase of 1 cm/s of RVs was associated with longer time onset of RV cardiomyopathy (HR 0.78, 95% CI 0.61–0.99) [9]. Right heart anatomic and physiologic echo measurements of tricuspid annular plane septal excursion (TAPSE) < 10 mm, RV fractional change < 20%, moderate to severe tricuspid regurgitation (TR), and pulmonary artery systolic pressure (PASP) > 50 mmHg have been associated with the reduced effectiveness of LBAP [68], but a direct causal relationship has not been definitively established. Reduced RV TAPSE has been shown to be predictive of RV dysfunction [60], but a TAPSE/PASP ratio (area under the curve (AUC) 0.761, 95% CI 0.62–0.90, *p* = 0.003) was better than isolated PASP (AUC 0.735, 95% CI 0.60–0.86, *p* = 0.002) or TAPSE (AUC 0.608, 95% CI 0.47–0.75, *p* = 0.140) in predicting heart failure in 70 patients undergoing CRT [69].

Valvular heart disease is becoming more appreciated as a contributor to and/or a sequela of LV dysfunction. Severe lead-induced TR is associated with an increased incidence of mortality and heart failure events [70,71]. In a study of 89 patients (age 64.1 ± 13.4 years) with none-to-mild TR (n = 58, 65.2%) or moderate-to-severe TR (n = 31, 34.8%) receiving LBAP, lead tricuspid annulus ≤ 16.1 mm, measured during ventricular end-diastole in the apical 3–4 chamber view, was independently associated with worsening TR after LBAP (HR 0.20, 95% CI 0.06–0.76, *p* = 0.017) over 19.0 ± 6.5 months follow-up [72]. In a small prospective cohort of 21 patients, transesophageal echocardiogram (TEE)-guided pacemaker or implantable cardioverter defibrillator lead implantation did not show worsening TR, whereas in the 103 consecutive historical control patients whose lead was implanted under fluoroscopy experienced worsening TR by 1 grade, (0% vs. 6.8%, *p* = 0.60). Although this investigation was limited by a small number of subjects and was not randomized, the findings suggest that perhaps transthoracic echocardiogram (TTE)-guided implantation potentially could reduce the future onset of TR [73]. With the advancement and availability of tricuspid valve repair, routine assessment of the progression TR secondary to lead impingement of a tricuspid valve leaflet can provide opportunities to optimize medical therapies before the onset and progression of severe RV dysfunction and right heart failure.

There is a limited body of evidence implicating that RV pacing may lead to atrial functional mitral regurgitation [74,75]. In a small study of 21 patients examining the impact of conduction system pacing in addition to CRT, mitral regurgitation was worse in those with RV pacing alone [76]. Further studies examining the temporal relation of PICM and left ventricular and left atrial functional mitral regurgitation are warranted.

2DE and 3DE are ideal for pacemaker pre-implantation planning, intra-procedural guidance of lead placement, and post-implantation optimization of electromechanical pacing and surveillance of development of left and right heart failure. The 2023 Heart Rhythm Society guideline on cardiac physiologic pacing for the avoidance and mitigation of heart failure recommends that TTE be performed within 3–12 months of a CRT or CSP device to assess LVEF in patients with decreased LVEF [3].

### 6.2. Multi-Parametric Echocardiographic Analysis of Pacing and Early Prediction of Cardiomyopathy

Mechanical dyssynchrony is defined as disparity in the timing of regional contraction or as uncoordinated, non-homogenous, regional myocardial motion [77]. It may be identified through septal flash, apical rocking, septal-to-posterior wall motion delay (SPWMD), or through various techniques utilizing longitudinal strain. Clinical application of these markers is more reliable in patients in normal sinus rhythm. Robust data validating these measurements in the setting of atrial fibrillation are lacking.

#### 6.2.1. Septal Flash and Apical Rocking

Septal flash, originally described as “septal beaking” on an M-mode echocardiography in patients with LBBB, is a short inward motion of the septum prior to LV ejection, reversing the usual left to right septal activation, occurring mostly during the isovolumetric period, which is followed by stretching of the LV lateral wall [78]. It may be assessed through visual identification 2DE or via M-mode echocardiography.

Apical rocking, initially identified as “apical shuffle” [79], is defined as abnormal septal to lateral rocking of the ventricular apex and may be related to intraventricular conduction delays (such as LBBB), regional damage (such as myocardial scar related to infarction), or some interplay of both. It can be visualized in an apical 4 chamber view as an early septal motion at the cardiac apex with a predominant lateral motion during ejection and demonstrated on cardiac strain imaging.

Apical rocking and septal flash frequently co-exist in patients with conduction delays, though apical rocking may be distinguished from the septal flash in that it occurs during the ejection phrase of the cardiac cycle and primarily affects the apex rather than the interventricular septum [80]. Both septal flash and apical rocking are frequently seen in RV pacing modalities.

#### 6.2.2. Septal-to-Posterior Wall Motion Delay

While septal flash and apical rocking may be visually striking, efforts to quantify mechanical dyssynchrony have subsequently been developed. One of the earliest was SPWMD, which is obtained via M-mode recording from the parasternal short axis at the papillary muscle level. SPWMD > 130 msec is indicative of LV dyssynchrony [81], and patients with SPWMD > 130 msec following pacemaker implantation are at increased risk of developing PICM over the next several years [82].

#### 6.2.3. Speckle-Tracking Strain

STE has seen a marked expansion in clinical utility over the past decade and, in 2025, is highly recommended for integration into clinical practice [83]. Nonetheless, there remain questions as to the utility of STE in prognostication post pacemaker implantation. Some promising uses of STE for prediction of PICM include mechanical propagation delay (MPD), standard deviation of time to peak strain dispersion (TPS-SD), and global longitudinal strain (GLS).

MPD is the average difference in time to peak strain between adjacent segments. Calculation of septal wall MPD in msec is performed via longitudinal strain as follows: [(mid septum − apical septum) + (basal septum − mid septum)]/2. Septal wall apex to base MPD > 50 msec measured several months after RV apical pacing was associated with a reduction in LVEF (sensitivity 81%; specificity 88%; AUC 0.84; 95% CI 0.75–0.91; *p* < 0.0001) [84].

Post-implantation GLS is a robust tool for the prediction of PICM, as shown in Table 2. Several small registries and observational studies have confirmed a reduction in GLS as an independent predictor of developing PICM in multivariate analysis. The average GLS measured 1 month after implantation of a pacemaker is significantly lower in the 15 patients who subsequently developed PICM (defined as a decrease in LVEF ≥ 5%) compared with those who did not (n = 40) (GLS −12.6% versus −16.4%, respectively, *p* = 0.022). One-month GLS had high predictive accuracy for determining the subsequent development of PICM (AUC 0.80, optimal GLS threshold: <−14.5%, sensitivity 82%, specificity 75%), and particularly PICM (AUC 0.86, optimal GLS threshold: <−13.5%, sensitivity 100%, specificity 71%) [84].

Another small study performed a similar analysis comparing patients who had a significant decrease in GLS (n = 18; baseline of −17.2% to −12.6%) at one month to those with a less substantial reduction (n = 42; baseline −17.5% to −16.4%). GLS values remained stable from month 1 to month 12, and the initial reduction in GLS was associated with a statistically significant increase in development of PICM [85].

Similarly, a larger single center prospective registry of 71 patients in Korea with preserved LVEF and high-grade AVB performed echocardiographic evaluation < 7 days after pacemaker implantation and followed patients with serial echocardiograms for 1 year. A GLS < −15.0% in the week following pacemaker implant had 100% sensitivity and 80.9% specificity for predicting PICM in the following year, with a receiver operating characteristic analysis demonstrating an AUC of 0.92 [86]. In addition to the absolute value of the GLS, the change over time can be predictive of PICM. For example, 37% of patients who had a decrement in GLS of ≥15% after RV pacemaker implantation subsequently had a >10% decrease in LVEF over 2 years of follow-up compared with just 12% of subjects who had <15% change in GLS (*p* = 0.004) [87].

In the setting of an abnormal strain, it may be valuable to include an assessment of peak strain dispersion (PSD), which is calculated as the time to peak strain measured from the onset of the Q wave to the peak negative strain throughout the cardiac cycle; a PSD > 60 msec is defined as dyssynchrony. Several groups have noted PSD to be indicative of cardiac dyssynchrony though a definitive link to PICM remains elusive [75,88,89].

Perhaps most helpful is evidence suggesting that a baseline GLS performed prior to pacemaker implantation can predict PICM. A retrospective analysis of 80 patients in Korea found an increased risk of PICM over 1 year in patients with GLS ≤ −20.7% HR 1.25 (95% CI 1.009–1.492, *p* = 0.004). The sensitivity and specificity for this value were 81% and 58%, respectively, with an AUC of 0.714 (*p* = 0.009) [90]. Noteworthy is that a larger retrospective analysis of 110 patients did not find the baseline GLS to be an independent predictor of PICM [87]. Hence, a post-implant echocardiogram demonstrating an absolute GLS < −14.5% or a significant reduction (≥15%) in GLS from baseline are likely more predictive than the strain obtained prior to pacemaker implantation.

Despite the strong predictive and prognostic value of GLS in PICM, its utility has not been well studied in patients with underlying atrial fibrillation. The absolute GLS value must be interpreted with caution because, in both paroxysmal and persistent atrial fibrillation, GLS has been demonstrated to be lower compared with normal sinus rhythm [91,92,93]. In addition, the irregular rhythm can contribute to variable and imprecise measurements. In this subgroup, a relative change in GLS may offer a more dependable assessment.

#### 6.2.4. Aortopulmonary Ejection Delay

The majority of echocardiographic tools to prognosticate PICM are measures of intraventricular dyssynchrony; however, interest exists in the right ventricle and potential effects of interventricular dyssynchrony. Although some measurements of the function are directly implicated in the development of PICM [9], the measurement of aortopulmonary ejection delay can provide additional evidence of interventricular dyssynchrony and is associated with an increased risk of developing PICM in multivariate analysis. An aortopulmonary ejection delay > 40 msec is considered abnormal [94].

### 6.3. Clinical Synthesis

Following the implantation of a pacemaker, an echocardiographic assessment may be performed, which can integrate a variety of subclinical markers that may predict a higher-than-average risk of developing PICM. The markers of intraventricular mechanical dyssynchrony include the visual clues of septal flash or apical rocking, the M-mode measurement of SPWMD, and the use of STE to determine GLS and MPD. Additionally, aortopulmonary delay may be determined. These parameters are summarized in Table 3. In patients with several indicators consistent with dyssynchrony, closer clinical monitoring may be indicated to identify patients who require resynchronization therapy.

### 6.4. Echocardiographic Signatures of Various Pacing Modalities

The various pacing modalities, including RVAP, RVSP, HBP, LBAP, and CRT, all have various echocardiographic signatures that can be interpreted and are summarized in Table 4.

#### 6.4.1. Right Ventricular Apical Pacing and Right Ventricular Septal Pacing

Approximately half of patients who undergo RV pacing will demonstrate some visual appearance of mechanical dyssynchrony, approximately one-quarter will demonstrate septal flash alone, and one-quarter will demonstrate both septal flash and apical rocking [95]. These visual findings are common regardless of whether the pacing lead was in the RV apex or the RV septum. Additionally, RV pacing will cause a SPWMD > 130 msec in approximately one-half of patients [94]. Pacemaker-induced reduction in LVEF may even be present acutely in patients who undergo RVAP or RVSP [96,97], and these may be harbingers of sustained or further reductions in LVEF.

Studies consistently demonstrate that GLS declines significantly in RV-paced patients compared with normal controls [96,98,99]. There is, however, heterogeneity in the effect of RVAP versus RVSP in GLS decline, with some studies demonstrating greater abnormality with RVAP than RVSP [98], and some demonstrating an equivalent decline in measured GLS [99]. Importantly, leadless pacemakers appear to have similar impact on GLS and LVEF as conventional RVAP or RVSP [100]. There are some markers of dyssynchrony that are reported in RVAP but not in RVSP. These include PSD and aortopulmonary ejection delay [94,97].

RV mechanics are an emerging area of interest in patients with RV pacing. Studies in this area are relatively small and short-term; however, an echocardiographic and physiologic picture is emerging that seems to favor RVSP. A randomized, single center study from Spain also found more evidence of mechanical dyssynchrony (48.1% versus 19.4%; *p* = 0.04) in patients with RVAP than RVSP; however, these findings did not translate into a statistically significant increase in PICM [101]. Finally, there are emerging data on left atrial strain parameters. RVSP appears to conserve left atrial strain in all three phases (reservoir, conduit, and contractile) compared with a modest decrement in left atrial reservoir and contractile strain in subjects who have RVAP [102]. Overall, the echocardiographic parameters of RVAP and RVSP seem more similar than different, which seems to translate into overall similar rates of developing PICM.

#### 6.4.2. Conduction System Pacing

CSP, including LBAP and HBP, generally shares similar echocardiographic signatures and is comparable to CRT in many ways. Notably, a prospective study of 151 patients undergoing LBAP demonstrated preserved GLS and LVEF over 2 years and that none of the patients developed PICM [66].

LBAP generally demonstrates an absence of echocardiographic signs of dyssynchrony and preserved LVEF over the mid to longer term, and measures of LV dyssynchrony are generally absent [103]. GLS is usually unchanged over the course of months to years [102]. Additionally, studies of LBAP have demonstrated the preservation of RV size and function (TAPSE, RV fractional area change), as well as RV free wall strain [53,97,104]. LBAP also demonstrates an improved biventricular synchronization compared with RVAP or RVSP [97,104,105,106]. Finally, left atrial strain is preserved in LBAP in all three phases [102].

HBP similarly demonstrates the preservation of GLS and left atrial volume index when compared with RV pacing [107], and importantly, there is no significant difference in the echocardiographic signature of selective versus non-selective HBP in terms of LVEF, left ventricular outflow tract velocity time integral, E/e’, LV GLS, LV pre-strain diameter, or classic measures of RV systolic function (i.e., TAPSE, RV S’) [89].

### 6.5. Clinical Synthesis

The current evidence is consistent in that mechanical dyssynchrony occurs in the early phase of PICM as measured by echocardiography is associated with an increased risk of progression to the development of definitive PICM in patients with initially preserved LVEF and who require frequent pacing. CSP avoids the echocardiographic signatures of dyssynchrony and appears to markedly reduce, if not eliminate, the risk of PICM in the short to intermediate term.

### 6.6. Magnetic Resonance Imaging

Cardiac MRI has emerged as a feasible alternative in the risk stratification of patients and can help identify patients who are at risk of developing PICM. It offers a unique mix of high intrinsic spatial and temporal resolution, allowing for the accurate quantification of volumes and LVEF, regional wall motion assessment, coupled with the characteristics of gadolinium contrast media, which can be used for the quantification of scar from both ischemic and non-ischemic processes [108]. The ability of MRI to provide tissue characterization is a unique feature and has been studied in the risk prediction of pacer-induced cardiomyopathies [109]. The detailed assessment of cardiac and extra cardiac anatomy is another advantage of cardiac MRI that might be useful in certain selections of patients.

#### 6.6.1. Assessing Dyssynchrony with Cardiac Magnetic Resonance Imaging

Despite the many advantages of MRI, the adoption of this technique has remained limited due to long scan times, poor image quality in the presence of intracardiac devices, and access [108]. An observational study of 84 patients with LVEF > 40% and the presence of a dual chamber pacemaker who underwent cardiac MRI pre- and post-implantation showed that the initiation of RV pacing in patients with fibrosis, quantified by cardiac MRI, was associated with an increase in LV end-systolic volume (5.3 ± 3.5 versus 2.1 ± 2.4 mL/m^2^; *p* < 0.01) and decreased EF (−5.7 ± 3.4% versus −3.2 ± 2.6%; *p* = 0.02) compared with those without fibrosis. After 6 months of RV pacing, 20% of patients had a decline in LVEF < 35% and were eligible for an upgrade to CRT. All patients with decline in LVEF had fibrosis on pre implant cardiac MRI. These findings suggest that fibrosis on cardiac MRI may be used to identify those at risk of developing heart failure before pacemaker implantation [110].

Assessing dyssynchrony with cardiac MRI has been well described in the literature in patients with CRT [111]. Three cardiac MRI techniques have been used for dyssynchrony measurement: myocardial tagging, displacement encoding with stimulated echoes (DENSE), and tissue velocity mapping (TVM). However, currently there is insufficient evidence to support the use of these cardiac MRI variables to predict the risk of PICM in patients undergoing RV pacing for high degree AVB and normal LVEF.

#### 6.6.2. Cardiac Magnetic Resonance in Assessing Response to Cardiac Resynchronization Therapy Versus Left Bundle Area Pacing

Several studies have examined the role of cardiac MRI in predicting the response to CRT or LBAP in patients with preexisting cardiomyopathy who are eligible for CRT [112,113]. The presence and extent of scar on pre-implant gadolinium-enhanced cardiac MRI correlated with post-implantation reverse remodeling [112]. The presence of transmural septal scar was associated with higher rates of procedural failures in LBAP [114] and correlated with worse outcomes. CRT responsiveness was assessed by the reduction in end-systolic volume or the improvement in LVEF compared with baseline. Patients with low scar burden demonstrated higher responsiveness to CRT [115]. An RCT of 102 patients with cardiomyopathy who underwent CRT evaluated the optimal strategy for LV lead placement using multi-modality imaging, combining strain echocardiography, cardiac MRI, and cardiac CT. There was no significant difference in the primary end point of end-systolic volume reduction (>15%) post-implant or in the clinical outcomes between the two groups [113]. These results are contradictory to other observational studies that suggested that delayed enhancement cardiac MRI may have a role in predicting CRT responsiveness. Extrapolating these data to patients with no structural heart disease and normal LVEF who undergo pacemaker implantation is challenging due to the lack of studies using cardiac MRI as a risk predictor for PICM in this population. This may be an area for future research, as access to CMR is increasing and scan times are reducing given technological improvements.

#### 6.6.3. Cardiac Magnetic Resonance Imaging and Myocardial Viability

Cardiac MRI plays an important role in the assessment of myocardial viability in patients with ischemic heart disease and heart failure who are considered for surgical revascularization. The percentage of wall thickness replaced by scar, quantified by late gadolinium enhancement (LGE) on cardiac MRI, is a sensitive marker that predicts the recovery of the LV contractile function after revascularization. The presence of transmural or >75% thickness LGE is associated with a very low probability of myocardial contractile function recovery in the involved segments [116]. On the contrary, the absence of LGE or <50% thickness LGE is associated with a high likelihood of myocardial function recovery post-revascularization [117]. Although well-validated in ischemic cardiomyopathy, the role of myocardial viability assessment using CMR has not been studied for the risk prediction of PICM in patients without known ischemic heart disease. Determining the prognostic role of viability in PICM could be an area of future investigation, which may refine the current practice.

### 6.7. Computed Tomography Imaging

Advanced cardiac imaging, including CT scans, is rapidly evolving in the management of intracardiac devices [109]. The highest utilization of cardiac CT is for the assessment of anatomical structures such as left atrial appendage anatomy for percutaneous device closures or pulmonary vein anatomy prior to atrial fibrillation ablation. Like MRI, cardiac CT can offer volumetric quantification of ventricles, but radiation exposure makes it a less attractive option. No studies were identified that specifically assessed the role of CT in predicting risks of PICM. Currently, the data are insufficient to make any recommendations regarding the use of cardiac CT in this population.

## 7. Clinical Practice Recommendations

**Figure 2:**
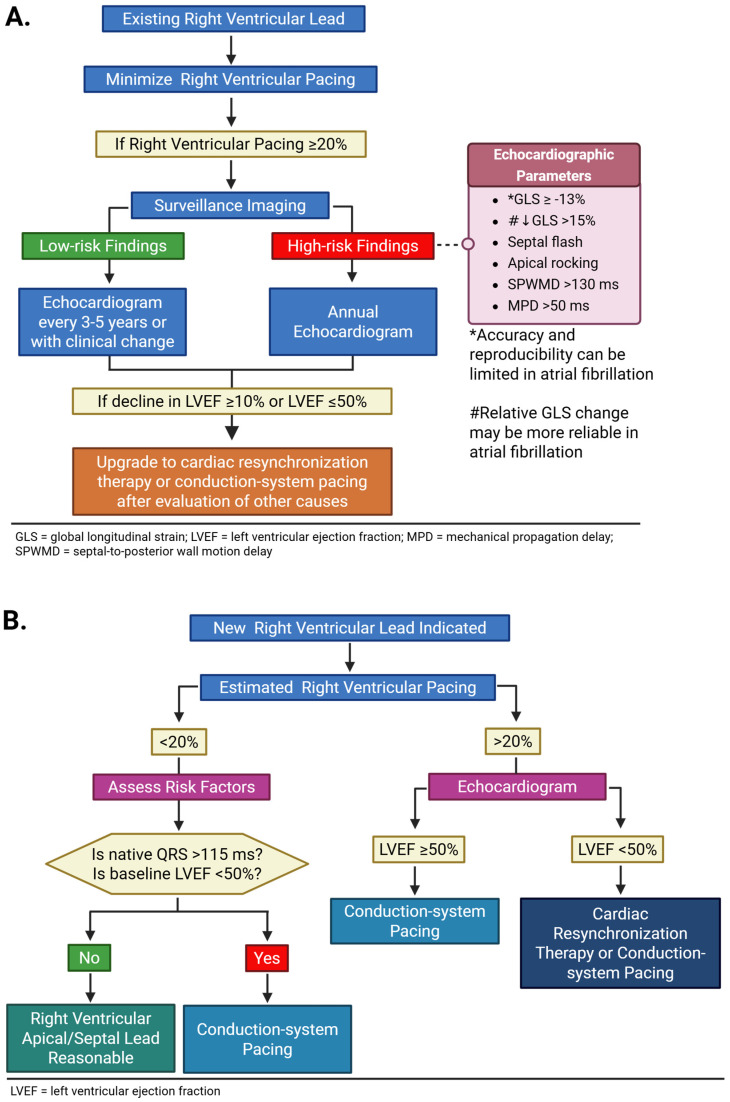
**Algorithm for image-guided surveillance and management of pacing-induced cardiomyopathy.** In patients with both existing right ventricular lead (**A**) and new right ventricular lead indication (**B**), the frequency of surveillance imaging monitoring is dependent on right ventricular pacing burden. Created in https://BioRender.com.

We summarize our clinical practice recommendations in Figure 2 and provide a novel approach utilizing imaging-based guidance for the risk assessment and surveillance of PICM (Table 5). In patients who are expected to require substantial ventricular pacing, a comprehensive assessment can help clinicians identify patients who are at higher risk of PICM to guide the initial pacing strategy (Figure 2A). The presence of any of the high-risk echocardiography-derived parameters has been associated with a higher likelihood of PICM development; however, the current evidence does not support prioritizing any marker because there are no direct comparison studies between these measurements. In addition, it is unknown if the number of abnormal markers directly correlates with the magnitude of LV systolic dysfunction. Nevertheless, absolute GLS value and relative GLS change are probably the most reliable and reproducible when the rhythm is normal sinus. A TTE with GLS, when available, and selective use of CMR may influence decision making. Patients with a low predicted risk may undergo conventional RV pacing, with intentional programming to minimize unnecessary ventricular pacing, whereas those with an intermediate to high risk of developing PICM should be considered for physiologic pacing (LBAP or CRT) rather than RV pacing. A retrospective study demonstrated increased in-hospital mortality in patients undergoing a CRT upgrade when compared with those patients undergoing a de novo CRT implant (1.9% versus 0.8%, *p* < 0.001) [118]. This study underscores that prevention is preferable to a later upgrade.

Following pacemaker implantation, early and longitudinal surveillance with echocardiogram should be performed to detect subclinical mechanical dyssynchrony and early decline in LVEF (Figure 2B). Global longitudinal and RV strain should be integrated into the basic echocardiographic examination protocol in the evaluation in patients with a paced rhythm. Imaging markers such as septal flash, apical rocking, septal-to-posterior wall motion delay, reductions in GLS, increased MPD, and prolonged aortopulmonary ejection delay should be reported. In patients with clear evidence of dyssynchrony, a risk-benefit discussion of upgrading pacing modality should be considered.

In summary, while the existing 2023 Heart Rhythm Society/Asia Pacific Heart Rhythm Society/Latin American Heart Rhythm Society included the use of echocardiography-derived LVEF to assess left ventricular function in patients with a paced rhythm, image-guided monitoring and treatment was not emphasized [3]. Similarly, recent reviews have also not focused on the central role of imaging in the management of PICM [1,16]. In this narrative review, we outlined the evidence supporting the contributions of prognostic echocardiographic and cardiac MRI markers in the detection of PICM. Particularly, in patients who undergo traditional (i.e., RVAP or RVSP; non-CSP) PPM, the best-available recommendation at this time based on limited but promising data is to obtain an echocardiogram 1 month post-implant and then annually in patients who demonstrate a significant (≥15%) reduction in GLS from baseline or an absolute GLS < −14.5% and are at increased risk of developing PICM in the next 1–2 years. The optimal timing of standard heart failure GDMT initiation for patients with normal LV systolic function, high RV pacing burden, and the presence of a high-risk echocardiographic profile is unknown. However, based on the best available data, we opine that it is reasonable to start monotherapy or a combination of ACEi, ARB, and β-blocker to maintain normal LV systolic function when the echocardiographic-derived surrogate markers of dyssynchrony are identified. Thus, if heart rate and blood pressure can accommodate, a trial of heart failure medications can be started after the 1-month or annual surveillance imaging is performed. Finally, cardiac MRI has an emerging role in the risk prediction of patients without structural heart disease undergoing pacemaker implantation. Although the presence of myocardial fibrosis on pre-implantation cardiac MRI was associated with a greater risk of developing PICM and the subsequent need for a CRT upgrade, the current data have not defined the optimal timing and frequency of MRI imaging in the prevention and treatment of PICM. Despite these limitations, there is currently sufficient evidence to consider the routine adoption of an imaging-directed approach in the management of PICM.

## 8. Future Directions

As PICM is more widely appreciated, clinical investigation is shifting from retrospective cohorts and case series to prospective and randomized trials aiming to determine optimal strategies to prevent and treat PICM. Several current studies comparing conduction system pacing and biventricular pacing in this patient population [44,119,120,121,122,123] are summarized in Table 6. Although some of these studies will provide insight into the role of imaging, especially echocardiography, there are no studies focused on the use of advanced cardiac imaging in the identification of those patients at risk. A knowledge gap also exists with respect to cardiomyopathy subgroups and various categories of atrial fibrillation. Thus, additional research is needed to validate the existing echocardiographic parameters and to identify novel imaging diagnostic and prognostic markers.

## 9. Conclusions

PICM is a potentially preventable complication of chronic and frequent RV pacing, driven primarily by electrical and mechanical dyssynchrony. Although traditional clinical and electrocardiographic risk factors remain useful, growing evidence demonstrates that cardiac imaging, particularly echocardiography with strain analysis, could identify subclinical dysfunction before clinical signs and symptoms of heart failure occur. The integration of imaging-derived risk markers into pre-implant planning and post-implant surveillance is essential in recognizing vulnerable patients and can lead to timely interventions.

## Figures and Tables

**Table 1:** Clinical risk factors for pacing-induced cardiomyopathy.

Clinical Risk Factors
**Pre-Implantation**
Older age
Male gender
History of myocardial infarction
Chronic kidney disease
Atrial fibrillation
Native QRS ≥ 115 ms
LVEF < 50%
**Post-Implantation**
Paced QRS ≥ 150 ms
Increased RV pacing burden ≥ 20%

LVEF = left ventricular ejection fraction; RV = right ventricular.

**Table 2:** Prediction of pacing-induced cardiomyopathy using global longitudinal strain.

Study	Pacing Modality	n	Key Predictive Parameter	Outcome
Ahmed, et al. (2014) [84]	RV apical pacing	55	1 month GLS < −14.5%	PICM onset at 1 year Sensitivity 82% Specificity 75% AUC 0.80 OR 24.77 (95% CI 1.17–344.70) *p* = 0.017
Datta, Dastidar, & Chakroborty (2023) [85]	RV apical pacing	60	1 month GLS < −14.5%	18/18 patients with GLS < 14.5% at 1 month developed PICM at 1 year
Kim, Park, & Jeong (2025) [86]	RV apical or RV mid-septal pacing	71	Post-implant GLS < −15.0%	PICM onset at 1 year Sensitivity 100% Specificity 80.9% AUC 0.92 OR 1.715 (95% CI 1.17–2.50) *p* = 0.005
Manocha, et al. (2023) [87]	RV apical pacing	110	Post-implant GLS ≥ 15% reduction	Increased risk (47% versus 16%; *p* = 0.001) of a composite of HF hospitalization, biventricular upgrade, or death over mean follow-up of 2 years. Increased risk (37% versus 12%; *p* = 0.004) of developing PICM.

AUC = Area under the curve; GLS = global longitudinal strain; OR = odds ratio; PICM = pacing-induced cardiomyopathy; RV = right ventricular.

**Table 3:** Echocardiographic parameters associated with elevated risk of pacing-induced cardiomyopathy.

Parameter	Safe Zone	At Risk
Septal Flash	Absent	Present
Apical Rocking	Absent	Present
Septal-to-Posterior Wall Motion Delay	***	>130 ms
Global Longitudinal Strain	≤−15%	≥−13%
Δ Global Longitudinal Strain	***	>15% decrement
Mechanical Propagation Delay	***	>50 ms
Aortopulmonary Ejection Delay	***	>40 ms

*** There are no defined values in the literature.

**Table 4:** Echocardiographic characteristics of different pacing modalities.

	Modality	RV Apical Pacing (RVAP)	RV Septal Pacing (RVSP)	Coronary Sinus (CS)/Cardiac Resynchronization Therapy (CRT) *	His Bundle Pacing (HBP)	Left Bundle Area Pacing (LBAP)
		Non-Physiologic (Muscle Capture)	Non-Physiologic (Muscle Capture)	Non-Physiologic (Muscle Capture)	Physiologic (Native Conduction)	Physiologic (Native Conduction)
Left Ventricular Mechanics	Septal Flash	Present	Present	Resolved	Absent	Absent
	Apical Rocking	Present	Present	Resolved	Absent	Absent
	Global Longitudinal Strain	Reduced	Reduced	Improved	Preserved	Preserved
Right Ventricular Mechanics	RV Systolic Function	Markedly Reduced	Reduced (relatively preserved compared to RAVP)	Variable	Preserved	Preserved
	RV Strain	Reduced	Reduced	Improved	Preserved	Preserved
Atrial Mechanics	Left Atrial Strain	Reduced	Preserved	Improved	Preserved	Preserved

* Indicated for patients with moderately reduced left ventricular ejection fraction. RV = right ventricular.

**Table 5:** Management of pacing-induced cardiomyopathy.

		Medical Therapy		Device Therapy	
Metric	PICM Definition	Preventive	Adjunctive	At-Risk for PICM	Established PICM
LVEF threshold	LVEF < 50% (most common) LVEF < 40% or <45% (less common)	Start ACEi, ARB, and β-blocker in patients with anticipated high RV pacing burden Greatest risk reduction with combination therapy	ACEi and ARB therapy. - Greater improvement in LVEF when combined with CRT/CS Atorvastatin, SGLT2 inhibitors, or MRAs - Limited data	Minimize RV pacing - Extend AV delay - Atrial-only pacing CSP or CRT in high-risk subgroup	Minimize RV pacing - Extend AV delay - Atrial-only pacing Upgrade device to CRT or CSP
∆ LVEF	↓ LVEF ≥ 10% (most common) ↓ LVEF ≥ 5% (less common)				
∆ RV function	≥2 abnormal parameters of RV systolic function				
RV pacing burden	≥20%, Budapest Trial, (most common) ≥40%, DAVID Trial, (less common)				

ACEi = angiotensin-converting enzyme inhibitor; ARB = angiotensin receptor blocker; AV = atrioventricular; CRT = cardiac resynchronization therapy; CSP = conduction system pacing; LVEF = left ventricular ejection fraction; MRA = mineralocorticoid receptor antagonist; PICM = pacing-induced cardiomyopathy; RV = right ventricular; SGLT2 = sodium–glucose co-transporter-2 inhibitor.

**Table 6:** Ongoing pacing-induced cardiomyopathy prevention and treatment trials.

Trial	Design/Population	Intervention	Primary Endpoint
DEEP Trial (NCT06474819) [119]	Patients need first pacemaker Normal/mildly reduced LVEF Expected high pacing burden	Randomized RV apical pacing vs. left septal/deep septal pacing	Incidence of PICM during first year post implantation
LEAP-CAR Trial (NCT05910866) [120]	Patients with advanced AV block Baseline LVEF > 45%	Randomized Left bundle branch area pacing vs. conventional RV pacing	Difference in LVEF on echocardiography at 12 months
LEAP Trial (NCT04595487) [121]	Patients with AV conduction disease Expected ≥ 20% ventricular pacing LVEF ≥ 40%	Randomized Left ventricular septal pacing vs. conventional RV pacing	Composite endpoint: all-cause mortality, heart failure hospitalization, and LVEF reduction > 10% at 1 year
Left vs. Left Randomized Clinical Trial (NCT05650658) [44]	Patients with LVEF ≤ 50%. QRS ≥ 130 ms Anticipated pacing burden > 40%	Randomized His or left bundle branch pacing vs. conventional biventricular pacing	All-cause death Hear failure hospitalization
His–Purkinje System Pacing vs. CRT in PICM (ChiCTR2000034265) [122]	Patients with established PICM following chronic RV pacing	Randomized Upgrade to His–Purkinje conduction system pacing vs. CRT	QRS complex duration. LVEF NT-proBNP
Preserve-Synch (DRKS00037554) [123]	Patients with AV block and expected high pacing burden LVEF > 40%	Randomized Left bundle branch area pacing vs. conventional RV pacing	Δ Global longitudinal strain PICM incidence Hear failure hospitalization CRT upgrade over 12 months.

AV = atrioventricular; CRT = cardiac resynchronization therapy; DEEP = left septal or deep septal pacing to orevent pacing-induced cardiomyopathy; LEAP = LVSP vs. RVP in patients with AV conduction disorders; LEAP-CAR = left bundle branch area pacing to avoid pacing-induced cardiomyopathy; LVEF = left ventricular ejection fraction; NT-proBNP = N-terminal pro B-type natriuretic peptide; PICM = pacing-induced cardiomyopathy.

## Data Availability

No datasets were generated or analyzed during the current study.

## Supplementary Materials

The following supporting information can be downloaded at: https://www.mdpi.com/article/10.3390/jcm15041358/s1, Supplementary Table S1 summarizes the characteristics and outcomes of pharmacotherapy trials conducted in patients who were at risk for or who have established PICM.
