# Supplementary material for: Advanced Cardiac Imaging for Risk Prediction of Pacing-Induced Cardiomyopathy: A Narrative Literature Review
Source: J Clin Med. 2026 Feb 9;15(4):1358. doi: 10.3390/jcm15041358 (PMC12942639; doi:10.3390/jcm15041358)
Supplement: Supplementary file 1 [file jcm-15-01358-s001.zip › jcm-4099520-supplementary.pdf]

Supplementary Table S1. Summary of the characteristics and outcomes of pharmacotherapy trials conducted in patients who were at risk for or who have established PICM.

Trial	Study Type	n	Study Population	Study Methods	PICM Definition	Outcome
Schwerg, 2015 [48]	Retrospective cohort<br><br>Screening	20	*Inclusion:*<br>Patients presenting for annual, routine pacemaker interrogation with ≥90% right ventricular pacing<br><br>3 months of optimal medical therapy vs CRT for patients with PICM<br><br>*Exclusion:*<br>Patients with single-chamber pacemaker<br><br>Patients with other etiologies of impaired LVEF<br><br>Age: 73.1±12.4 years<br><br>Women: 47%	Screened patients with ≥90% right ventricular pacing<br><br>PICM patients received 3 months of optimal medical therapy<br><br>CRT upgrade if symptomatic left ventricular dysfunction<br><br>Follow-up: 3, 6, 12 months	Preserved left ventricular function at first pacemaker implantation and LVEF <45% unexplained by other cardiac disease with right ventricular pacing ≥90%	LVEF improved significantly from 33.3±5.2% pre-upgrade to 47.6±9.3% within 6 months post-upgrade (p<0.001)<br><br>Optimal medical therapy did not affect LVEF
Barbieri, 2002 [49]	Prospective<br><br>Post hoc analysis	54	*Inclusion:*<br>Symptomatic heart failure with LVEF <40% despite optimal	Transthoracic echocardiography performed prior to implantation and 3-5	Symptomatic heart failure with LVEF <40% despite optimal medical therapy and	Previous treatment with heart failure medications did not

			medical therapy and right ventricular pacing >40%<br><br>Adjusted device programming to minimize right ventricular pacing prior to enrollment<br><br>PICM patients who received CRT	months after CRT activation<br><br>Evaluated effect of heart failure medication (BB, ACEi/ARB, MRA) on CRT	right ventricular pacing >40%	reduce CRT upgrade incidence<br><br>CRT response (↓ LVESV by >15%) achieved in 30 patients (55.6%)
Lin, 2022 [51]	Single center<br><br>Retrospective cohort	1717<br><br>Statin: 240<br><br>Nonstatin: 1477	*Inclusion:*<br>Mobitz type II or complete atrioventricular block with pacemaker implantation<br><br>*Exclusion:*<br>Patients with alternative etiologies of heart failure or taking statin > 6 months<br><br>Age:<br>Statin - 73.9±10.5 years<br>Nonstatin - 73.8±5.0 years<br><br>Women:	Randomized to statin or nonstatin<br><br>Primary outcome: (cardiovascular death or heart failure hospitalization)<br><br>Secondary outcomes: Heart failure hospitalization<br>All-cause mortality<br>Cardiovascular death<br><br>Follow-up:<br>Statin - 5.1±3.3 years<br>Nonstatin - 5.3±3.5 years	↓ LVEF ≥10%<br><br>LVEF <50% post pacemaker implantation<br><br>CRT upgrade after exclusion of other etiologies of cardiomyopathy	Statin was associated with ↓ cardiovascular death or heart hospitalization (HR 0.69 [95% CI 0.56-0.84], p<0.001)<br><br>Statin was associated with ↓ heart hospitalization (HR 0.45 [95% CI 0.30-0.67], p<0.001)<br><br>Statin was associated with ↓ all-cause mortality (HR 0.52 [95% CI 0.45-0.61], p<0.001)<br><br>Statin was associated with ↓ cardiovascular

			Statin: 55%<br>Nonstatin: 54%			death (HR 0.65 [95% CI 0.49-0.86], p=0.003)
Kerley, 2023 [50]	Single center<br><br>Retrospective cohort	43	*Inclusion:*<br>PICM patients who had CRT upgrade from a single- or dual-chamber pacemaker from 2011-2021<br><br>*Exclusion:*<br>Patients with alternative etiologies of cardiomyopathy<br><br>Age: 77±10.8 years<br><br>Women: 30.2%	Measured LVEF pre- and post-upgrade at 3 to 6 months<br><br>Identified LVEF improvement using multivariate linear regression<br><br>Follow-up: 24 months	↓ LVEF ≥10% with >20% right ventricular pacing burden and with prior LVEF ≥50% prior to or immediately after pacemaker implantation<br><br>Severe PICM: LVEF <35%	LVEF significantly improved from 28.7% pre- upgrade to 44.3% post-CRT upgrade (p<0.01)<br><br>CRT response (↑LVEF >5%) observed in 88.4% of patients<br><br>Trend of ACEi/ARB use with LVEF improvement (↑ by 7.21%, p=0.05)
Agha, 2025 [45]	Single center<br><br>Retrospective cohort	642	*Inclusion:*<br>Adult patients with normal LVEF and complete heart block who had single- or dual-chamber pacemaker with right ventricular lead implantation<br><br>*Exclusion:*<br>Patients who received epicardial, coronary sinus, or His bundle area leads	Comparison of medical therapy group (ACEI/ARB, BB, or both) vs control group<br><br>Fine-Gray subdistribution hazard models (treated death as a competing risk)<br><br>Follow-up: maximum 10 years	Upgrade to a biventricular device or reduction in↓ LVEF to ≤40% without another cause	Risk of PICM: significantly lower in medical therapy group (HR 0.59 [95% CI, 0.45-0.77]) vs control group<br><br>Lowest risk in patients on combination therapy (ACEI/ARB and BB) (HR 0.46 [95% CI 0.31-0.69])

			Pre-existing cardiomyopathy or reduced LVEF before implantation<br>Ischemic cardiomyopathy during follow-up<br><br>Age: 71±14 years<br><br>Women: 51%			

ACEi = angiotensin-converting enzyme inhibitor; ARB = angiotensin receptor block; BB = β-blocker; CI = confidence interval; CRT = cardiac resynchronization therapy; HR = hazard ratio; LVEF = left ventricular ejection fraction; MRA = mineralocorticoid receptor antagonist; PICM = pacing-induced cardiomyopathy
